# The Rate of Nonallelic Homologous Recombination in Males Is Highly Variable, Correlated between Monozygotic Twins and Independent of Age

**DOI:** 10.1371/journal.pgen.1004195

**Published:** 2014-03-06

**Authors:** Jacqueline A. L. MacArthur, Timothy D. Spector, Sarah J. Lindsay, Massimo Mangino, Raj Gill, Kerrin S. Small, Matthew E. Hurles

**Affiliations:** 1 Wellcome Trust Sanger Institute, Wellcome Trust Genome Campus, Hinxton, Cambridge, United Kingdom; 2 Department of Twin Research and Genetic Epidemiology, King's College London, St. Thomas' Hospital, London, United Kingdom; University of Oxford, United Kingdom

## Abstract

Nonallelic homologous recombination (NAHR) between highly similar duplicated sequences generates chromosomal deletions, duplications and inversions, which can cause diverse genetic disorders. Little is known about interindividual variation in NAHR rates and the factors that influence this. We estimated the rate of deletion at the *CMT1A-REP* NAHR hotspot in sperm DNA from 34 male donors, including 16 monozygotic (MZ) co-twins (8 twin pairs) aged 24 to 67 years old. The average NAHR rate was 3.5×10^−5^ with a seven-fold variation across individuals. Despite good statistical power to detect even a subtle correlation, we observed no relationship between age of unrelated individuals and the rate of NAHR in their sperm, likely reflecting the meiotic-specific origin of these events. We then estimated the heritability of deletion rate by calculating the intraclass correlation (ICC) within MZ co-twins, revealing a significant correlation between MZ co-twins (ICC = 0.784, p = 0.0039), with MZ co-twins being significantly more correlated than unrelated pairs. We showed that this heritability cannot be explained by variation in *PRDM9*, a known regulator of NAHR, or variation within the NAHR hotspot itself. We also did not detect any correlation between Body Mass Index (BMI), smoking status or alcohol intake and rate of NAHR. Our results suggest that other, as yet unidentified, genetic or environmental factors play a significant role in the regulation of NAHR and are responsible for the extensive variation in the population for the probability of fathering a child with a genomic disorder resulting from a pathogenic deletion.

## Author Summary

Many genetic disorders are caused by deletions of specific regions of DNA in sperm or egg cells that go on to produce a child. This can occur through ectopic homologous recombination between highly similar segments of DNA at different positions within the genome. Little is known about the differences in rates of deletion between individuals or the factors that influence this. We analysed the rate of deletion at one such section of DNA in sperm DNA from 34 male donors, including 16 monozygotic co-twins. We observed a seven-fold variation in deletion rate across individuals. Deletion rate is significantly correlated between monozygote co-twins, indicating that deletion rate is heritable. This heritability cannot be explained by age, any known genetic regulator of deletion rate, Body Mass Index, smoking status or alcohol intake. Our results suggest that other, as yet unidentified, genetic or environmental factors play a significant role in the regulation of deletion. These factors are responsible for the extensive variation in the population for the probability of fathering a child with a genomic disorder resulting from a pathogenic deletion.

## Introduction

Homologous recombination (HR), leading to crossing over and exchange between homologous DNA sequences, occurs during meiosis and ensures that each gamete contains a unique mixture of maternal and paternal DNA. Occasionally HR occurs ectopically between highly similar duplicated sequences or paralogous genomic segments, such as segmental duplications, in a process known as non-allelic homologous recombination (NAHR). NAHR between directly oriented duplicated sequences on the same chromosome gives rise to a chromosomal deletion, and, if it occurs in a inter-molecular fashion, can generate a reciprocal duplication on the other chromosome, whereas NAHR between duplicated sequences in an inverted orientation leads to inversions. The breakpoints of NAHR rearrangements cluster in defined hot spots within segmental duplications that reflect hotspots of HR activity [1].

Genomic rearrangements resulting from NAHR can be manifested as genomic disorders, predominantly due to the altered copy number of dosage-sensitive genes [2], or non-pathogenic structural variation [3]. Several NAHR hotspots have been identified due to their association with specific genomic disorders, with reciprocal deletion and duplication events being associated with different disorders at some loci [4]–. For example NAHR between two copies of the *CMT1A-REP* segmental duplication on 17p12 leads to deletion of a 1.4 Mb region including the *PMP22* gene resulting in hereditary neuropathy with liability to pressure palsies (HNPP), with reciprocal duplication of the same region resulting in Charcot-Marie-Tooth disease type 1A (CMT1A) [8].

Until recently the rates of rearrangement at any particular locus were estimated from the frequency of the resultant dominant disease phenotype in the population. It is now possible to estimate the frequency of recombination in males through direct analysis of sperm by PCR amplification of breakpoint products [9]. Direct analysis of rates of rearrangement in germline DNA, at four NAHR hotspots, revealed variation in rate both between individuals and loci [9], however very little is known about the extent of the interindividual variation or the factors which influence this.

Both genetic and non-genetic factors have been suggested to play a role in influencing mutation rates. It has previously been shown that variation in *cis* can influence the rate of chromosomal translocations [10], [11]. Several properties of duplicated sequences have been shown to be major determinants of the rate of nonallelic homologous recombination, with rate increasing with length and sequence similarity and decreasing with distance between repeats [12], [13]. Recently *PRDM9* has been identified as a genome wide *trans* regulator of meiotic recombination in humans and mice [14]–[18], and variation within this gene has been shown to significantly alter rates of meiotic recombination and instability, including *CMT1A-REP* rearrangements [15]. Recombination rate is also correlated with density of the recombination hotspot motif [12], [17] to which PRDM9 binds [14].

Evolutionary and epidemiological studies have suggested that mutation rates are higher in the paternal germline and increase with paternal, but not maternal, age. Thus age is an important potential confounder to consider in any investigation of genetic and environmental influences on mutation rate. These studies are consistent with the observation that the male germline entails greater numbers of mitotic replications than the female germline, and that the number of paternal mitotic replications increases with age, whereas the number of maternal mitotic replications does not [19]. However, most studies on this topic have focused on base substitutions and not structural variants. Two previous studies could not detect an age dependent effect for *de novo* deletions and duplications flanked by duplicated sequences [20], [21], although they observed conflicting results for events not flanked by duplications [20], [21]. Moreover, there was little evidence of a genome-wide parent of origin bias for NAHR deletions and duplications from either study [20], [21]. However, a significant paternal origin bias has been observed at the *CMT1A-REPs* specifically [22]. The presence or absence of a paternal age effect for a given mutational process may depend on whether the underlying mutation mechanism is dependent on, or potentiated by, mitotic replication. It has previously been shown that NAHR at four different NAHR hotspots, including the *CMT1A-REP*s, is specific to meiosis [9].

To further investigate the role of age, genetic variation and environmental factors on interindividual variation in rates of NAHR we directly estimated the rate of deletion at the *CMT1A-REPs* in sperm samples from 34 UK males from an extensively phenotyped twins cohort, for which we had determined their *PRDM9* genotype. These samples included 8 pairs of MZ twins, which allowed us to demonstrate a significant role for shared genetic variation and/or shared environment in determining rate of NAHR in sperm.

## Results

### Age has no effect on NAHR rate at the *CMT1A-REP* hotspot

**Figure 1:**
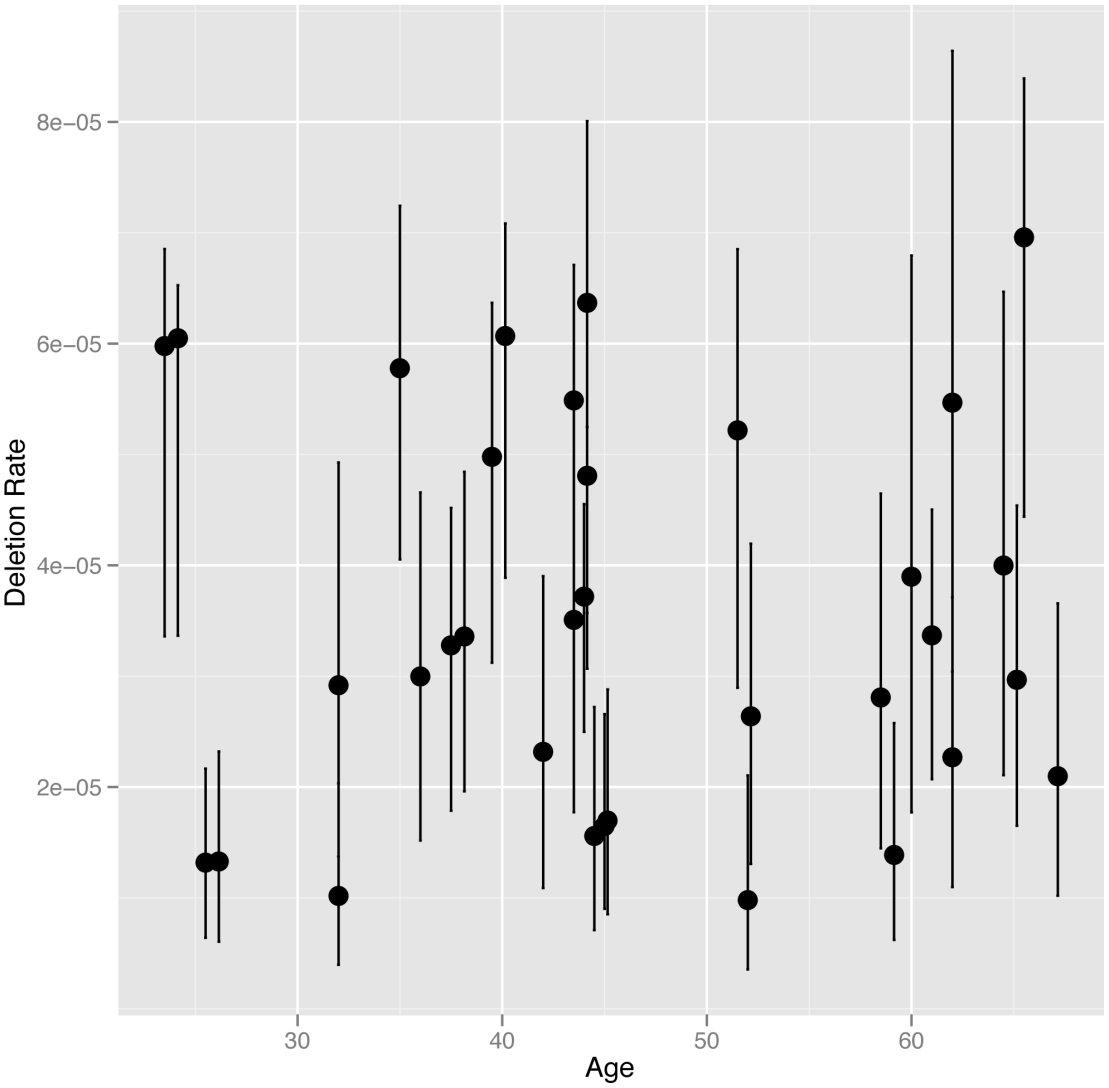
NAHR deletion rate at the *CMT1A* locus plotted against age at the time the sample was provided. In cases where multiple individuals have the same age the points are plotted slightly offset from each other on the x-axis. The 95% confidence intervals for each estimate of deletion rate are shown.

We directly estimated the rate of NAHR generated deletions at the *CMT1A-REP* hotspot in sperm DNA from 34 UK males, including 16 monozygotic (MZ) co-twins (8 twin pairs), six dizygotic (DZ) co-twins (3 twin pairs) and 12 unrelated individuals, using the assay described previously [9] (results shown in Table S1). The *CMT1A-REP* deletion was chosen for analysis in this study as it has the highest rate of NAHR among loci that can be robustly assayed, and therefore the lowest measurement error, as well as the ease of resequencing the NAHR hotspot in different individuals. The average rate of deletion in our cohort of 3.54×10^−5^ (+/−3.04×10^−6^ s.e.m), with a range of 9.82×10^−6^ to 6.96×10^−5^, is consistent with the rate (4.20×10^−5^) reported previously [9]. We analysed deletion rate as a function of the age of the individual at the time the sperm sample was produced (24 to 67 years)(Figure 1). No age dependent increase in deletion rate was observed among males, with linear regression analysis of deletion rate on donor age showing no correlation (R^2^ = 5×10^−4^, p = 0.9019).

It has been suggested that the paternal mutation rate increases with age due to an increase in the number of germ-cell divisions, and therefore genome replications, to complete spermatogenesis. The number of genome replications occurring during spermatogenesis in a man of a certain age can be estimated as follows: 30 prior to puberty, 23 spermatogonial stem cells replications per year after puberty, followed by 5 during sperm maturation [19]. Assuming puberty occurs at age 15 a sperm produced by a man of age 24 will have undergone 242 genome replications, while a sperm produced by a man of age 67 will have undergone 1231. This equates to a 5.1 fold linear increase in the number of genome replications between ages 24 and 67 (the ages of the youngest and oldest donors in our study). To test whether we have the statistical power to detect such a fold change with our data we performed simulations to assess the power of our study design to detect a paternal age effect should it exist (as described in Material and Methods). Our simulations showed that we had 100% power to detect a fold change of 5.1 between our youngest and oldest donors, and had 95% power to detect a much smaller fold linear change of 1.7 (Figure S1). We also used simulations to exclude a non-linear paternal age affect similar to that observed between maternal age and prevalence of Down syndrome [23]. Thus we can confidently exclude that even a weak linear or non-linear paternal age effect exists for NAHR at this locus.

### NAHR deletion rate at *CMT1A* is heritable

**Figure 2:**
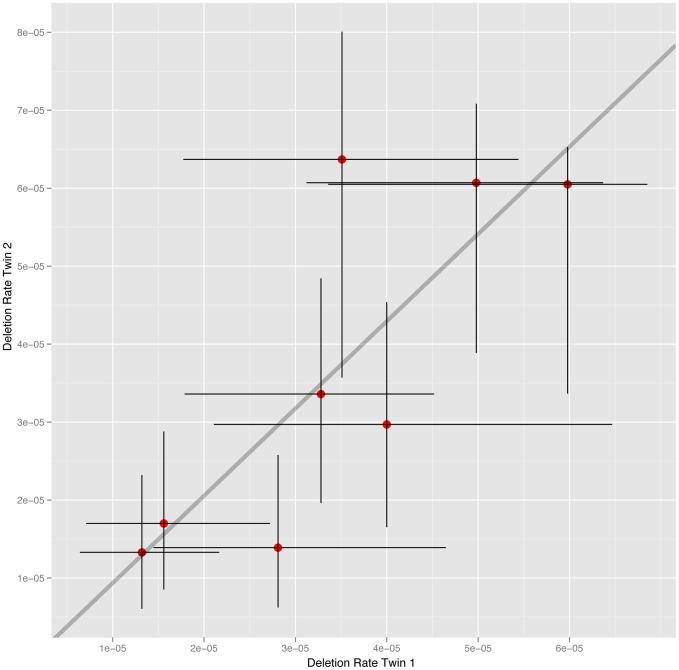
NAHR deletion rate at the *CMT1A* locus in MZ co-twins. The estimates of deletion rate for each co-twin are plotted against one another, with 95% confidence intervals for each estimate also shown.

The cohort of 34 males studied includes 16 MZ co-twins (8 twin-pairs). To estimate the heritability of deletion rate we calculated the intraclass correlation (ICC) within MZ co-twins and unrelated MZ pairs. The estimate of deletion rate for each MZ co-twin plotted against one another is shown in Figure 2. The analysis reveals a significant correlation between MZ co-twins (ICC = 0.784, 95% CI 0.292–0.952, p = 0.0040). In order to test whether the MZ twins are more highly correlated than unrelated pairs we randomly sampled 8 pairs of unrelated individuals from the MZ co-twins, calculated the ICC and p-value for the generated sample set, and repeated this 10,000 times. Out of the 10,000 simulated unrelated sample sets only 64 (0.64%) had an ICC greater than that observed in the MZ co-twins and a p-value less than that observed in co-twins. The rate of NAHR in MZ co-twins is therefore significantly more similar than in unrelated pairs of individuals. The observed heritability is due to the rate of deletion being determined by elements shared by MZ co-twins, namely genetics or shared environment. Comparing the level of heritability observed between MZ and DZ co-twins is often used to distinguish between the relative effects of these elements on inherited traits. MZ co-twins share 100% of their genetic variation, compared to 50% on average shared between DZ co-twins, while MZ and DZ twins are expected to have a similar level of shared environment. Due to the limited number of DZ co-twins available in this study (6 co-twins, 3 twin-pairs) we are unable to use the direct comparison between MZ and DZ co-twins to distinguish between the relative effects of genetics and shared environment on deletion rate, despite repeat attempts to sample sperm from additional DZ co-twins.

However in the vast majority of traits explored in this twin cohort any estimated effect of shared environment is usually much less than 5% of the variance.

### Investigation of genetic determinants of *CMT1A-REP* deletion rate

In an attempt to identify specific genetic or environmental factors involved in the regulation of deletion rate we investigated the effect of possible determinants of NAHR. Variation in the gene *PRDM9* is a known genetic determinant of NAHR rate, with variation at the *PRDM9* zinc finger having been shown to regulate recombination including NAHR deletion rate at *CMT1A* [15]. We cloned and sequenced the *PRDM9* zinc finger alleles from each individual, and aligned these to published sequences [14], [15] to classify the alleles as described previously. The majority of individuals in our cohort were genotyped as A/A homozygote (29/34, 85%), with 4 A/B heterozygotes and one A/L20 heterozygote. A/A homozygotes exhibited high levels of variability in deletion rate, with a seven-fold range of 9.82×10^−6^ to 6.96×10^−5^. The high level of variability observed within A/A homozygotes means that we can exclude variation at *PRDM9* as being a major cause of the correlation observed between MZ twins.

**Figure 3:**
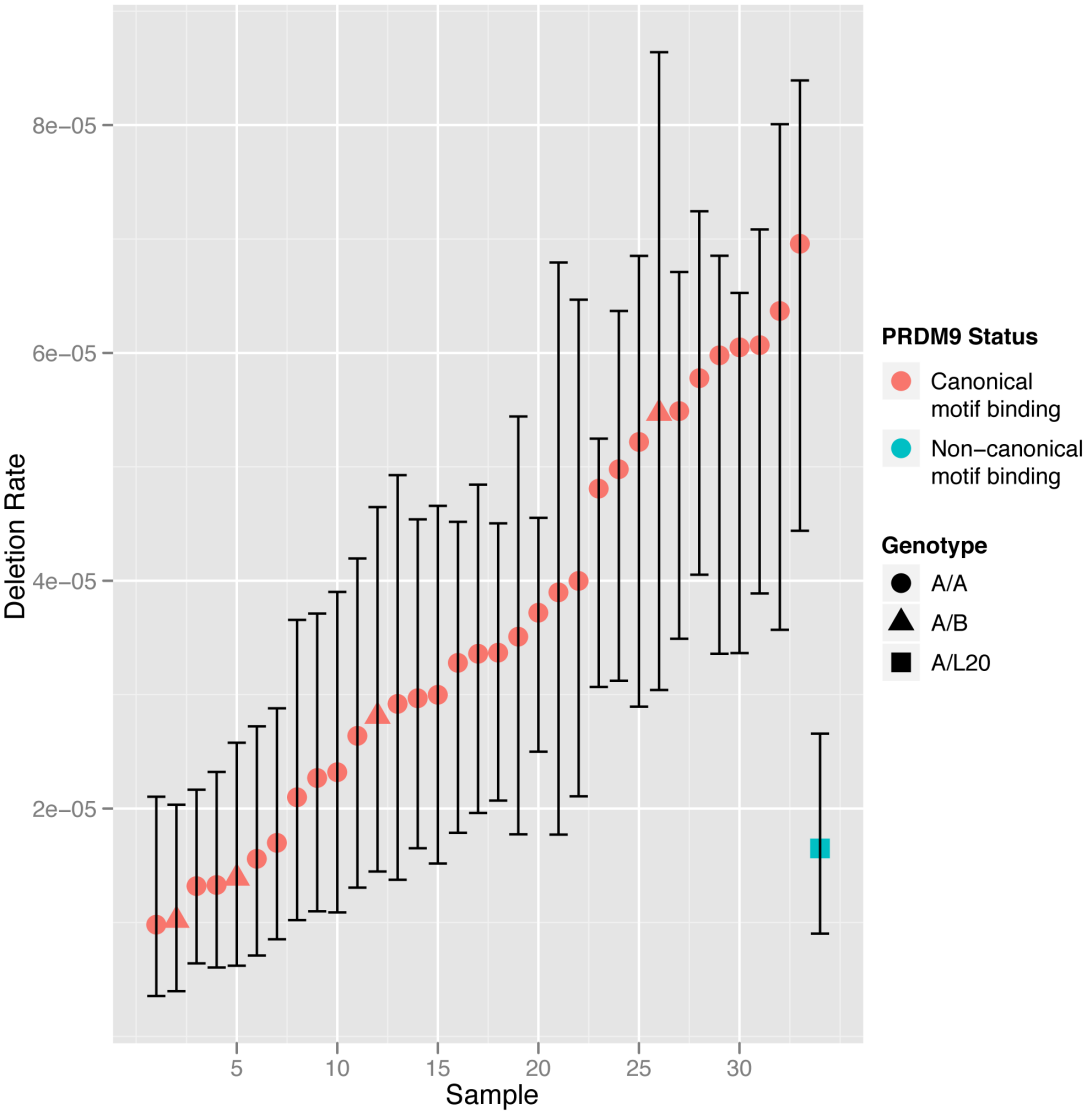
NAHR deletion rate at the *CMT1A* locus in all the sperm samples analysed with men grouped according to *PRDM9* zinc finger motif-binding status. Men homozygous for alleles recognising the canonical motif (shown in red) or heterozygous for alleles recognising the canonical and non-canonical motifs (shown in blue) are grouped separately and shown in ascending order. The 95% confidence intervals for each estimate of deletion rate are shown.

We classified the different minor *PRDM9* alleles that we observed into those predicted to bind to the canonical recombination hotspot motif, and those predicted a bind a non-canonical motif. Only the L20 allele present in a single male is predicted to bind a non-canonical motif. The deletion rate in the A/L20 sample falls within the range observed in the samples homozygous for alleles recognising the canonical motif, although it is towards the lower end. We also found no significant differences in NAHR rate between the males homozygous and heterozygous for the A allele (p = 0.1104, Mann-Whitney test). The deletion rate for each twin arranged by *PRDM9* motif binding classification is shown in Figure 3.

We also tested for association of the rate of deletion at the *CMT1A-REP*s with 4 SNPs at 3 loci (rs1670533, rs3796619, rs17542943, rs7863596) previously shown in genome-wide association studies to have modest effects on rates of allelic recombination [24], [25], in 13 unrelated semen donors for whom data were available from genome-wide SNP genotyping chips. Unsurprisingly, given the small sample size and known modest effect sizes we observed no significant (p<0.05) association after Bonferoni correction for multiple testing.

Sequence similarity between duplicated sequences has been proposed to be one of the primary determinants of NAHR rate, along with the length of the duplicated sequences, the distance between the duplicated sequences and the density of HR hotspot motifs [26], [27]. We sequenced the proximal and distal *CMT1A-REP* NAHR hotspots in one of each of the eight MZ co-twins. We tested for association between the average NAHR rate in these 8 co-twins and sequence similarity between proximal and distal hotspots but we observed no significant association (p = 0.27, linear regression), suggesting that variation in local sequence similarity is not a primary determinant of NAHR at this hotspot. In addition, we did not observe any size variation in the PCR amplifications of the proximal and distal segmental duplications, suggesting that in the males studied here there are no sizeable structural variants within the *CMT1A-REP* but outside the NAHR hotspot.

### There is no evidence that Body Mass Index (BMI), smoking or alcohol intake are determinants of *CMT1A* deletion rate

The heritability of *CMT1A* deletion rate could be due to elements in the shared environments of MZ co-twins that effect recombination rate. Studies have shown that several environmental mutagens affect mutation rate and could have direct effects on NAHR rate. Analysis of human populations exposed to radiation has revealed that germline mutation rates are increased by ionizing radiation [28], while studies in animals have revealed that several environmental pollutants increase mutation rate in the male germline [29]–[32]. Several lifestyle choices have also been identified as having potential mutagenic effects through their association with cancer [33], while sperm of smokers have been shown to have higher frequencies of DNA damage [34].

We were able to obtain information on BMI, smoking status and alcohol intake for a subset of our twin samples. We did not detect any correlation between deletion rate and BMI (R^2^ = 0.06, p = 0.2357, linear model) in an analysis of 27 twin samples. Reliable information on alcohol intake and smoking status at the time of sampling was only available for ten samples. No correlation was detected between deletion rate and alcohol intake (R^2^ = 0.03, p = 0.6385, linear model) or smoking status, irrespective of whether ex-smokers are classified as smokers (p = 0.9143, Mann-Whitney) or non-smokers (p = 0.7111, Mann-Whitney). However due to the small sample size for which smoking status and alcohol intake data was available we are unlikely to have the power to detect any subtle correlations between these risk factors and deletion rate.

## Discussion

In this study we have demonstrated that there is no appreciable paternal age effect on NAHR recombination rate at the *CMT1A-REP* locus. This raises the question of why a paternal age effect is observed for some mutation processes and not others. The absence of an age effect in this process is likely due to its meiotic-specific nature. Paternal age has been most strongly linked to the rate of base substitution [35], which may occur due to errors in DNA replication. As we have described the number of DNA replications to complete spermatogenesis increases with age. Previous studies detected no paternal age effect on translocation [36] or allelic recombination [37]–[39] rates, which are both meiotic in nature. The number of meiotic divisions involved in spermatogenesis is fixed at two and does not increase with age. These observations suggest that germline mutation events that are observed to exhibit an increase in frequency with paternal age are predominantly mitotic in nature, while those that fail to exhibit such an effect are meiotic. These observations are supported by the observation that patients with pathogenic *de novo* deletions and duplications mediated by NAHR do not have significantly older parents than matched controls [20]. It is also worth noting that paternal age effects could also be affected by the positive selection and clonal expansion of cells in the testes, but this is typically linked to a very small number of specific activating mutations [40].

We have detected a significant correlation in NAHR deletion rate at the *CMT1A* locus between MZ co-twins, with MZ co-twins being significantly more correlated than unrelated MZ pairs. MZ co-twins are essentially genetically identical, are the same age and share many aspects of their environment. Any of these shared effects could be determinants of heritable traits. In our analysis we have excluded age, variation at the *PRDM9* zinc finger and variation in sequence similarity between paralogous hotspot sequences as being the cause of the observed heritability of NAHR rate. Therefore we conclude that the observed heritability is due to the effects of genetics, shared environment or a combination of these factors.

The mutation process we are analysing occurs during meiosis in the male germline. As the only two meiotic divisions in spermatogenesis occur immediately before sperm formation the deletion events we are analysing will have occurred only a few weeks prior to the sample being provided. It therefore seems likely that among different types of shared environmental factors, those that relate to lifestyle choices or habits in adulthood may play more of a role than shared childhood environment. Although it is also possible that shared environment in childhood could induce epigenetic effects that extend into adulthood.

We did not detect any correlation between BMI, smoking status or alcohol intake with deletion rate in analysis of a subset of our twin samples, however for smoking status and alcohol intake the small number of these samples mean we only had the power to detect a substantial effect. Further investigation is required into the possible environmental determinants of deletion rate. Due to the previously reported links between smoking and genetic aberrations in sperm [34] we see particular value in extending this study to investigate the effects of smoking on NAHR in a larger number of samples.

Our results suggest that a large fraction of the heritability in deletion rate between MZ co-twins may be due to shared genetic variation. Although *PRDM9* has been identified as a significant regulator of NAHR, variation at the *PRDM9* zinc finger cannot explain the heritability of recombination rate observed in this study. There are likely to be additional genetic factors, with either genome-wide or locus-specific effects, which determine the rate of deletion at the *CMT1A* locus. Several genome-wide association studies have set out to identify genetic determinants of allelic recombination through pedigree analysis of crossovers [24], [25], [41]. Several loci have been identified that are associated with allelic recombination in males including a two single-nucleotide haplotype in the *RNF212* gene [24], [25], [41], in addition to SNPs at 7q36.1 (nearest gene *NUB1*) and 9q31.3 (nearest gene *UGCG*) [24]. Rnf212, Ugcg and Nub1 expression is induced at meiosis with a peak at diplotene stage of prophase 1, which corresponds to chromosome chiasmata resolution and supports their potential roles during meiosis [24]. It should be noted that different loci are associated with recombination rate in males and females; the *RNF212* haplotype that is associated with high recombination rate in males is associated with low recombination rate in females [24], [41]. It is possible that these genome wide determinants of allelic recombination also affect NAHR rate, although their modest effect sizes on allelic homologous recombination means that they are highly unlikely to explain the seven-fold range in deletion rates observed in our study.

It is possible that locus-specific regulators of NAHR rate exist. This hypothesis is supported by observations that polymorphisms within the regions flanking the segmental duplications involved in Williams-Beuren Syndrome increase the rate of NAHR [10]. Locus specific regulators of mutation rate have also been identified at the *NID1* meiotic recombination hotspot [42], minisatellite *MS32* [43] and within the palindromic repeats involved in t(11;22) translocation [11], [44]. Locus specific regulators of NAHR rate at *CMT1A-REP*s may exist, however none have been identified to date. Repeat length, sequence similarity and distance between repeats have also been shown to influence NAHR [12], [13], along with concentration of a hotspot motif [12], [17]. We did not detect any correlation between sequence similarity at the proximal and distal *CMT1A-REP* NAHR hotspots and NAHR rate, and we did not observe any variation in repeat length. It seems likely that variation in distance between repeats and concentration of the hotspot motif is more likely to effect variation between loci than between individuals at this particular locus.

We expect that the phenomena of NAHR rate heritability and absence of paternal age effect that we observed in this study are generalisable to other loci subject to meiotic-specific NAHR. The *CMT1A-REP* NAHR hotspot is not an outlier with respect to the amount of variation in NAHR rates between individuals compared to other NAHR hotspots [9]. Indeed, if the primary determinants of this variation in rate are shared environmental factors or *trans*-acting genetic variation we may expect rates of NAHR to vary in a correlated fashion across many NAHR hotspots. Whereas if the primary determinants of rare variation are locus-specific in action (*e.g*. *cis*-acting genetic variation) rates of NAHR across loci in different individuals would be uncorrelated. We would also expect the same factors to influence rates of NAHR-generated duplications, as duplications and deletions are reciprocal products of the same event.

These findings necessitate further investigation into the genetic control of NAHR rate. An important next step would be to determine whether genetic control is genome-wide or locus-specific through the analysis of multiple hotspots within each individual. Such an analysis would be limited however by the number of sperm available from each individual and the labour intensive nature of this technique.

Given the pathogenic nature of the deletion we studied, these observations have striking clinical implications. They suggest that either there are potentially modifiable environmental factors that alter the probability of having a child with a genomic disorder, and/or different males vary markedly from the population average in their risk of having a child with a genomic disorder by virtue of variation in their genome. The population impact of these mutagenic factors will depend on whether they influence NAHR rate variation locus-specifically or genome-wide, and, with approximately half of pathogenic NAHR events coming from the maternal germline, whether they are specific to the male germline. While we estimated the average rate of NAHR-mediated deletion at the *CMT1A-REP* to be 1 in ∼30,000 sperm, the cumulative impact of pathogenic NAHR events genome-wide, and including both paternal and maternal germlines, is likely to be observed in more than 1 in 3,000 births [45].

## Materials and Methods

### Ethics statement

All samples and information were collected with written and signed informed consent. The study was approved by the St Thomas' Hospital Research Ethics Committee.

### Samples

Semen samples were obtained from 34 volunteers included in the TwinsUK adult twin registry, based at St Thomas' Hospital, King's College, London (www.twinsuk.ac.uk). The ages of these individuals at sampling ranged from 24 to 67 years. DNA samples were randomized and relabeled to enable estimation of deletion rate blinded to sample relatedness.

### DNA extraction from semen

DNA was extracted from semen samples using a protocol adapted from the QIAamp Tissue Protocol using the QIAamp DNA Blood Maxi Kit (Qiagen). 1 ml of semen was transferred to a 50 ml Falcon tube and 20 ml Buffer 1 (150 mM NaCl, 10 mM EDTA (pH 8.0)) added, before vortexing for 10 seconds and centrifuging at 4000 rpm for 10 minutes. The supernatant was discarded into Virkon disinfectant (Day Impex Ltd) and the pellet resuspended in 3 ml Buffer 2 (100 mM Tris·Cl (pH 8.0), 10 mM EDTA, 500 mM NaCl, 1% SDS, 2% β-mercaptoethanol). 400 µl Proteinase K was added to the solution and incubated at 55°C with gentle rocking or occasional inversion. After 2 hours an additional 100 µl Proteinase K was added and incubated for a further 2 hours at 55°C as before. 6 ml Buffer AL (Qiagen) was added to the solution and incubated for 10 minutes at 70°C, before adding 5 ml of Ethanol, followed by mixing by inverting the tube 10 times, then shaking. The solution was transferred onto the QIAamp maxi column (Qiagen) placed in a 50 ml centrifuge tube, taking care not to moisten the rim, the cap closed and centrifuged at 3000 rpm for 3 minutes. The filtrate was discarded and the QIAamp maxi column placed back in the 50 ml centrifuge tube. 5 ml of buffer AW1 was added to the QIAamp Maxi column, the cap closed, and centrifuged for 3350 g for 1 minute. 5 ml of buffer AW2 was added to the QIAamp Maxi column, the cap closed, and centrifuged at 3350 g for 15 minutes. The filtrate was discarded and the QIAamp column placed in a clean 50 ml centrifuge tube. 550 µl distilled water, equilibrated to room temperature, was pipetted onto the membrane of the QIAamp Maxi column, the cap closed and incubated at room temperature for 5 minutes before centrifuging at 3350 g for 2 minutes. This elution step was repeated to give a total elution volume of approximately 1 ml.

### Cloning and sequencing of *PRDM9* alleles

The sequence encoding the *PRDM9* zinc finger was amplified in 25 µl reactions using primers PRDM9_F3 and PRDM9_R1 [14] with final concentrations of 0.5 mM for each primer, 4.5 mM MgCl_2_, 0.05 U/ml of Taq polymerase/Pfu polymerase mix (10 units Taq:1 unit Pfu), and 1× PCR buffer system as described in [46] to amplify 25 ng of input DNA. Thermal cycling conditions were: 96°C for 20 seconds for one cycle, followed by 96°C for 10 seconds, 60°C for 20 seconds and 68°C for 2 min, for 30 cycles. Following gel electrophoresis bands were excised, DNA extracted using the QIAquick Gel Extraction kit (Qiagen) and cloned using the TOPO TA Cloning Kit for Sequencing (Invitrogen). Twelve colonies were picked from each transformation, cultured overnight and sequenced with primers 214F (TGATTGTTTCTTCATTTGATCTTCA), 731F (TGGAGAGTGTGGACAAGGTTT), 1742R (AGCAGAGGCTTGACCTATCG) and 1992R (GTCATGAAAGTGGCGGATTT) using 4∶1 Big Dye Terminator:dGTP Chemistry (Applied BioSystems).

### Estimating amplification efficiency

PCR efficiency was estimated for each sample by carrying out PCR reactions with approximately single molecule inputs of DNA. Each DNA sample was diluted to 20 pg/µl with 1 ng/µl herring sperm DNA. The *CMT1A* proximal repeat was amplified using primers CMT1A_PF1 and CMT1A_PR1 in the primary PCR and primers CMT1A_PF2 and CMT1A_PR2 in the secondary PCR [9]. PCR reactions were carried out as described previously to amplify *CMT1A-REP* deletion products with an input of 5 pg sample per reaction. A subset of positive amplification products were confirmed by reamplifying a subset of plates and showing 100% concordance with wells containing positive products, along with sequencing products from a subset of positive wells to confirm that all were consistent with the *CMT1A* proximal segmental duplication (data not shown). Poisson analysis was used to calculate the number of amplifiable molecules in each reaction using the equation –N ln[(N – R)/N], where N is the number of reactions performed and R is the number of positive reactions observed, and the mass of one haploid genome calculated for each sample.

### Estimation of *CMT1A* deletion rate

Amplification of *CMT1A-REP* deletion products was carried out as described in [9], but with 2 µl of diluted PCR product used as a template in the secondary PCR. Poisson correction of positive results and calculation of confidence limits was also carried out as described previously [9]. The number of input molecules in each well was estimated from the DNA input and the mass of one amplifiable haploid molecule, estimated by limiting dilution PCR for each sample. A subset of positive deletion products were confirmed by reamplifying a subset of plates and showing 100% concordance with wells containing positive products, along with sequencing products from a subset of positive wells (data not shown).

### Sequence analysis of *CMT1A-REP* hotspot

Primers (CCATGATCACCCTCATGTCA and CATGCAAACGAAAATGAAGC) were designed to amplify the hotspot for NAHR across the proximal and distal repeats.

PCR was carried out using REdAccuTaq LA DNA polymerase from Sigma Aldritch under the following conditions in a 50 µl volume, with approximately 25–50 ng of template DNA:

96° 30 secs, 94° 15 secs, 60° 30 secs −1° per cycle, 68° 3 min, go to step 2 6 times, 94° 15 secs, 57° 30 secs, 68° 3 min, go to step 6 29 times, 68° 10 mins, 4° hold.

After amplification, PCR products were ethanol precipitated and resuspended in 5–10 µl of double distilled water. Ligation and transformation into Pgem-TEasy (Promega) was carried out according to the manufacturers instructions. 48 clones were prepped for each individual, and 24 were sequenced.

Sequencing was carried out using standard dye-terminators and the external PCR primers. Internal sequencing primers were designed on each strand at +500 bp, +1000 bp and +1500 bp, respectively, so each clone was sequenced with a total of 8 primers.

Sequence analysis was carried out in GAP4[47]. Clones were assembled with the proximal and distal reference sequences and trimmed to the length of the PCR product. Clones were separated into proximal or distal specific contigs based on a stable 5 bp insertion in the distal repeat. Each sequence was manually inspected and base calling errors caused by dye-blobs and PCR errors were edited. Finally, the consensus sequence for the proximal and distal repeats was exported and aligned in SeaView4[48] using ClustalW2.

### Statistical analysis

Intraclass correlation was calculated in R [49] using the irr package (http://CRAN.R-project.org/package=irr) and 95% confidence intervals on the Poisson-corrected number of counts were calculated in R using the epitools package (http://CRAN.R-project.org/package=epitools). SNP association analyses were performed including only one sample per family by linear regression using deletion rate as a quantitative trait against individual SNP genotypes. In addition, we also performed a linear regression haplotye-based association analysis. Both analyses were performed using PLINK [50].

### Simulation of paternal age effect

We simulated a linear fold change in NAHR rate between ages 24 and 67 by setting the mean deletion rate in the simulated data to the observed mean deletion rate and estimating the expected rate for each sample, using their actual age, by assuming a linear increase from 24 to 67 years old, for different values of fold change from 1.1x to 10x. The measured rate of NAHR for each sample was simulated by random sampling from the Poisson distribution around the expected rate, and the correlation between age and simulated rates of NAHR was tested using linear regression, with a p-value threshold of 0.05. The proportion of replicates for which a significant result was achieved was equated to the statistical power of the experiment for that fold-change.

The observed increase in the prevalence of Down's syndrome with maternal age has been proposed to follow a logit logistic model [23]. We tested the power of our data to detect such a relationship between deletion rate and age by simulating a logit logistic curve from our data. The midpoint of the simulated deletion rates was set to the observed mean deletion rate in our data and a deletion rate for each sample generated by sampling from the Poisson distribution. A p-value for the correlation between the ages and simulated rates was calculated using Spearman's rank correlation test.

## Supporting Information

Figure S1 Power distribution for simulations of linear fold increases in deletion rate with age. Linear fold increases of between 1.1 and 10 in deletion rate between the ages of 24 and 67 were simulated as described. Each simulation was repeated 10,000 times, with the correlation between each simulated set of rates tested using the linear model, to give a p-value. The power represents the percent of simulations with a p-value below 0.05 for each fold change. The red line indicates a fold change of 5.1, the linear fold increase in the number of chromosome replications occurring during spermatogenesis between men of ages 24 and 67.
(DOCX)

Table S1 Summary data for all men analysed. Each man analysed is shown, coded by study number, along with age at the time the sperm sample was provided, zygosity (MZ = monozygotic, DZ = dizygotic), PRDM9 allele status, number of NAHR deletion molecules detected (Poisson corrected), estimated total number of molecules analysed and deletion rate at the CMT1A locus. Paired twins are indicated by sharing the same study number followed by either a _1 or _2.
(DOCX)
