# Supplementary material for: The Rate of Nonallelic Homologous Recombination in Males Is Highly Variable, Correlated between Monozygotic Twins and Independent of Age
Source: PLoS Genet. 2014 Mar 6;10(3):e1004195. doi: 10.1371/journal.pgen.1004195 (PMC3945173; doi:10.1371/journal.pgen.1004195)
Supplement: Table S1 — Summary data for all men analysed. Each man analysed is shown, coded by study number, along with age at the time the sperm sample was provided, zygosity (MZ = monozygotic, DZ = dizygotic), PRDM9 allele status, number of NAHR deletion molecules detected (Poisson corrected), estimated total number of molecules analysed and deletion rate at the CMT1A locus. Paired twins are indicated by sharing the same study number followed by either a _1 or _2. (DOCX) [file pgen.1004195.s002.docx]

| **Study number** | **Age (years)** | **Zygosity** | **PRDM9 alleles** | **Number of NAHR deletion molecules (Poisson corrected)** | **Estimated total number of molecules analysed** | **Deletion rate** |
| --- | --- | --- | --- | --- | --- | --- |
| 01_1 | 32 | DZ | A/A | 11.68 | 399445 | 2.92E-05 |
| 02_1 | 52 | DZ | A/A | 26.29 | 503670 | 5.22E-05 |
| 02_2 | 52 | DZ | A/A | 13.97 | 529904 | 2.64E-05 |
| 03_1 | 62 | DZ | A/B | 16.46 | 300711 | 5.47E-05 |
| 04_1 | 40 | MZ | A/A | 36.21 | 727725 | 4.98E-05 |
| 04_2 | 40 | MZ | A/A | 52.59 | 866206 | 6.07E-05 |
| 05_1 | 44 | MZ | A/A | 50.12 | 1347894 | 3.72E-05 |
| 06_1 | 35 | MZ | A/A | 51.69 | 894365 | 5.78E-05 |
| 07_1 | 60 | MZ | A/A | 10.56 | 270677 | 3.90E-05 |
| 08_1 | 42 | MZ | A/A | 11.68 | 504378 | 2.32E-05 |
| 09_1 | 61 | MZ | A/A | 30.26 | 898596 | 3.37E-05 |
| 10_1 | 65 | MZ | A/A | 14.54 | 363177 | 4.00E-05 |
| 10_2 | 65 | MZ | A/A | 17.80 | 599692 | 2.97E-05 |
| 11_1 | 45 | MZ | A/A | 10.56 | 675648 | 1.56E-05 |
| 11_2 | 45 | MZ | A/A | 12.39 | 727725 | 1.70E-05 |
| 12_1 | 66 | DZ | A/A | 46.12 | 662819 | 6.96E-05 |
| 12_2 | 67 | DZ | A/A | 11.33 | 538249 | 2.10E-05 |
| 13_1 | 45 | MZ | A/L20 | 15.40 | 931034 | 1.65E-05 |
| 14_1 | 44 | DZ | A/A | 43.60 | 794512 | 5.49E-05 |
| 14_2 | 44 | DZ | A/A | 67.63 | 1407342 | 4.81E-05 |
| 15_1 | 36 | MZ | A/A | 15.13 | 504378 | 3.00E-05 |
| 16_1 | 38 | MZ | A/A | 22.43 | 683760 | 3.28E-05 |
| 16_2 | 38 | MZ | A/A | 22.24 | 662819 | 3.36E-05 |
| 17_1 | 52 | DZ | A/A | 6.10 | 620690 | 9.82E-06 |
| 18_1 | 59 | MZ | A/B | 13.46 | 478401 | 2.81E-05 |
| 18_2 | 59 | MZ | A/B | 9.22 | 662819 | 1.39E-05 |
| 19_1 | 44 | MZ | A/A | 15.13 | 431647 | 3.51E-05 |
| 19_2 | 44 | MZ | A/A | 30.32 | 475778 | 6.37E-05 |
| 20_1 | 24 | MZ | A/A | 40.44 | 676243 | 5.98E-05 |
| 20_2 | 24 | MZ | A/A | 48.37 | 799118 | 6.05E-05 |
| 21_1 | 62 | MZ | A/A | 12.82 | 564706 | 2.27E-05 |
| 22_1 | 26 | MZ | A/A | 12.82 | 967831 | 1.32E-05 |
| 22_2 | 26 | MZ | A/A | 10.56 | 792332 | 1.33E-05 |
| 23_1 | 32 | MZ | A/B | 7.27 | 709360 | 1.02E-05 |
