# Supplementary material for: The Rate of Nonallelic Homologous Recombination in Males Is Highly Variable, Correlated between Monozygotic Twins and Independent of Age
Source: PLoS Genet. 2014 Mar 6;10(3):e1004195. doi: 10.1371/journal.pgen.1004195 (PMC3945173; doi:10.1371/journal.pgen.1004195)
Supplement: Supplementary file 2 [file pgen.1004195.s001.docx]

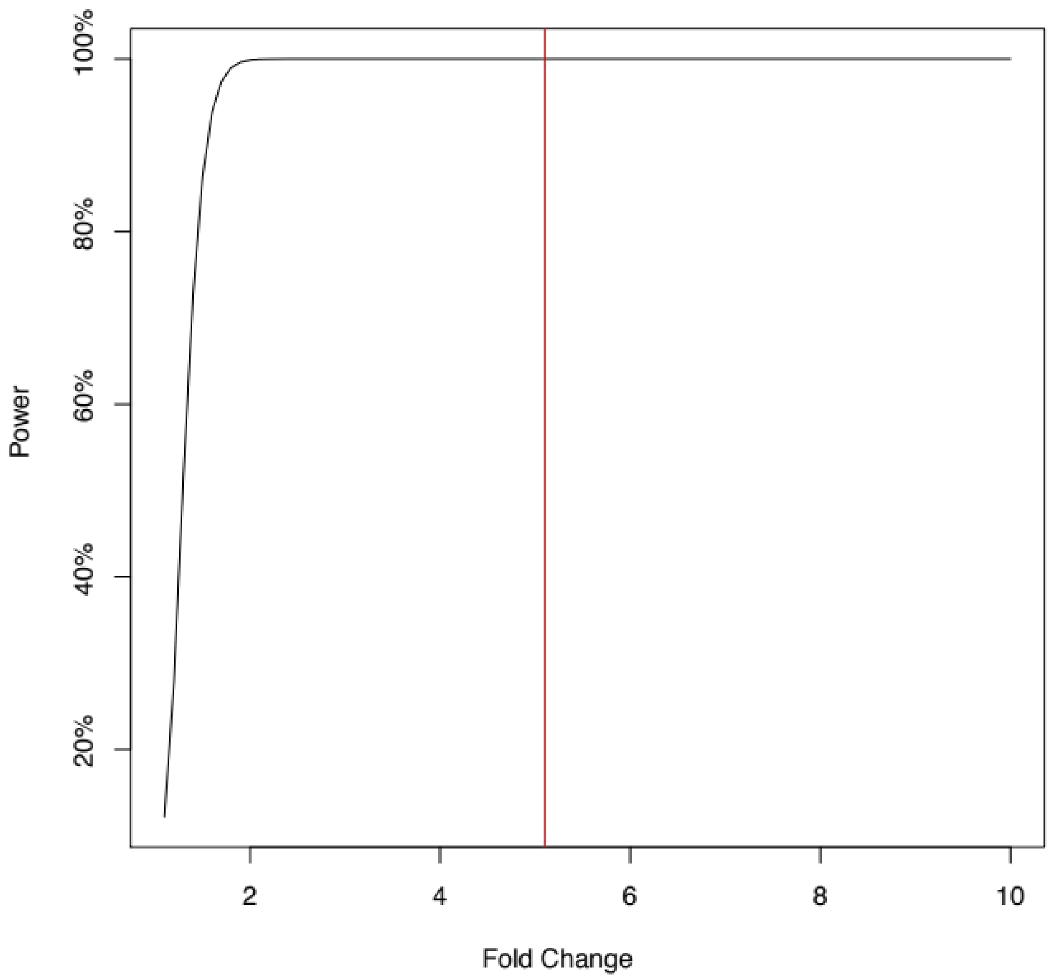

Power
100%
80%
60%
40%
20%
2
4
6
8
10
Fold Change
